# Nanopore-Aware Embedded Detection for Mobile DNA Sequencing: A Viterbi–HMM Design Versus Deep Learning Approaches

**DOI:** 10.3390/bios15090569

**Published:** 2025-09-01

**Authors:** Karim Hammad, Zhongpan Wu, Ebrahim Ghafar-Zadeh, Sebastian Magierowski

**Affiliations:** 1 Arab Academy for Science, Technology and Maritime Transport, Cairo P.O. Box 2033, Egypt; khammad@aast.edu; 2 Department of Electrical Engineering and Computer Science, Lassonde School of Engineering, York University, North York, ON M3J 1P3, Canada; symwanyy@yorku.ca (Z.W.); egz@yorku.ca (E.G.-Z.)

**Keywords:** DNA sequencing, RISC-V, edge computing, ARM, Rocket, nanopore sensing, Viterbi, FPGA

## Abstract

Nanopore-based DNA sequencing has emerged as a transformative biosensing technology, enabling real-time molecular diagnostics in compact and mobile form factors. However, the computational complexity of the basecalling process—the step that translates raw nanopore signals into nucleotide sequences—poses a critical energy challenge for mobile deployment. While deep learning (DL) models currently dominate this task due to their high accuracy, they demand substantial power budgets and computing resources, making them unsuitable for portable or field-scale biosensor platforms. In this work, we propose an embedded hardware–software framework for DNA sequence detection that leverages a Viterbi-based Hidden Markov Model (HMM) implemented on a custom 64-bit RISC-V core. The proposed HMM detector is realized on an off-the-shelf Virtex-7 FPGA and evaluated against state-of-the-art DL-based basecallers in terms of energy efficiency and inference accuracy. From one side, the experimental results show that our system achieves an energy efficiency improvement of 6.5×, 5.5×, and 4.6×, respectively, compared to similar HMM-based detectors implemented on a commodity x86 processor, Cortex-A9 ARM embedded system, and a previously published Rocket-based system. From another side, the proposed detector demonstrates 15× and 2.4× energy efficiency superiority over state-of-the-art DL-based detectors, with competitive accuracy and sufficient throughput for field-based genomic surveillance applications and point-of-care diagnostics. This study highlights the practical advantages of classical probabilistic algorithms when tightly integrated with lightweight embedded processors for biosensing applications constrained by energy, size, and latency.

## 1. Introduction

Biomolecular measurement technology has achieved impressive advances over the last decade [1]. This has been especially noteworthy in the case of DNA measurement, which is unlocking a new wave of applications—from point-of-care diagnostics, personalized medicine [2], and environmental biosurveillance [3] to remote disease outbreak monitoring and field-based genomic sensing [4]. The rapidly evolving nanopore sensors are a prime example for such technology that have enabled commercially available smartphone-sized sequencing devices capable of measuring the equivalent of a human genome in less than 10 h [5]. Their present ability to gather and digitize such measurements within a 1 W power window makes them potential targets for mobile scenarios as well. The ability of such devices to process DNA input on demand, in real time, also suggests amenability for deployment as remote sensors.

Repurposing such devices as ‘DNA meters’ on the ‘edge’ may have far-reaching positive impacts. As components in the IoT space, this includes the potential for affordable access to genetic analysis, rapid genomic surveillance, incorporation into industrial processes, and even adoption by information technologies that exploit molecular substrates [6]. However, the computational requirements of such DNA meters are of a fundamental concern, especially within mobile sequencing platforms. In particular, although today’s mobile DNA measuring machines can gather a significant amount of raw data, they lack any substantial internal computational ability to extract genomic information content. Rather, they transmit their measurements to external computers for downstream bioinformatic processing [7]. For many contemporary well-equipped genomic laboratories, this is sufficient. However, this approach is likely inadequate for operation in unstructured environments or field-based sequencing sites [8]. Hence, the development of an energy-efficient DNA computing engine co-located with front-end sequencing devices (i.e., similar to the vision recently presented in [9]) and suitable for portable battery-enabled sequencing devices is essential and *motivates* the technology proposed in this paper.

Although a number of computational steps follow DNA measurement, among the first and among the most computationally intensive of these is the base sequence detection (also known as *basecalling*). The task is responsible for converting the DNA molecule’s measured raw ionic current signal, from nanopores, to the text string equivalent (the *read*) of the nucleotide sequence. This task is computationally intensive and typically performed on external servers using deep learning (DL)-based algorithms, which—although accurate—consume tens of watts and demand substantial memory and compute infrastructure. Such requirements hinder deployment in mobile or energy-constrained environments, where data offloading is impractical or costly. Hence, without a loss of generality, the evolving mobile sequencing applications (i.e., the ultimate focus of this work) dictate the innovation of sequence detection hardware architectures characterized by high throughput, accuracy, and energy-efficient capabilities. Despite the numerous efforts reported in the literature in this space, a standard—albeit in a universal sense—hardware architecture that completely fulfills such conflicting requirements is not defined yet.

In this paper, we revisit the classical Viterbi-based Hidden Markov Model (HMM) as a viable and energy-efficient alternative to DL methods for nanopore basecalling. While DL models excel in inference accuracy, our results indicate that the accuracy gains may not justify their energy cost in scenarios where approximate basecalling suffices—e.g., bacterial strain detection, pathogen surveillance, and triage diagnostics. Our focus is on enabling embedded in situ DNA basecalling, directly co-located with the nanopore sensor, using ultra-low-power hardware that maintains sufficient accuracy and throughput for mobile operation. The following two subsections highlight these efforts to emphasize the specific motivation and contribution of the proposed framework.

### 1.1. Related Work

At present, sequence detection techniques require intensive computing requirements that employ machine learning algorithms. These are classified into DL and dynamic programming (DP) techniques. The DP technique for solving the sequence detection problem was first introduced in [10]. The proposed Viterbi-based HMM scheme in [10] demonstrated a promising performance for detecting an emulated DNA signal (as obtained from a solid-state nanopore sensor [11,12], which is described in more detail below). Following this introduction, various edge computing platforms have been reported in the literature [13,14] for the Viterbi detection scheme. In [13], a hardware-accelerated architecture, composed of an x86 CPU and FPGA accelerator, was presented for the detector outlined in [10]. In [14], this approach was generalized to hardware architecture scalable to detection algorithms of varying complexity. Despite the fact that both designs in [13,14] achieve remarkably high performance and energy efficiency, they do not offer a compact-sized architecture that fits palm-sized mobile sequencers. In particular, both studies emphasized the significant potential of adopting hardware accelerators in boosting the performance and energy efficiency of a classical laboratory DNA sequencing setup (in which the bioinformatic computing engine is implemented outside the sequencing device using off-the-shelf CPUs and/or GPUs) to levels suitable for mobile sequencers within a 6 W margin. Similarly, the CPU-only based Viterbi detector reported in [15] does not offer any path for a mobile sequencing platform in terms of size and power. On the other hand, the first embedded Viterbi framework for the DNA sequence detection problem was recently presented in [16]. The authors adopted the emerging RISC-V Rocket core [17] to execute the sequence detection program on a Spartan-6 FPGA device operated and controlled by Xilinx Zedboard ZNQ7020. The detector showed an energy efficiency improvement of 1.95× and 1.38× compared to other CPU and ARM implementations, respectively, with a power budget of 100 mW.

It is also worth mentioning that various deep learning neural network (DNN) architectures have been widely studied in the literature for the DNA basecalling problem. In most cases, this class of basecallers has demonstrated a remarkable inference accuracy to their HMM counterparts. However, the high accuracy for NN basecalling engines comes at the expense of more complex architectures with demanding hardware resources and power consumption levels. In particular, the recent study reported in [18] showed that the effective power (while neglecting the idle power of the computing platform) consumed by the DeepNano-coral detector (i.e., convolutional NN (CNN) based) ranges from 10 W to 92 W on a desktop workstation and 9 W on a laptop workstation. The study has also reported a minimum of 48 W consumed by another popular recurrent NN (RNN) detector, known as Guppy. Other studies like [19,20] have considered improving the computational efficiency of the basecalling problem indirectly by adopting a pre-filtering stage for raw nanopore signals. The associated DNN basecalling architecture in [19] ran within a power budget of 28 W. Such early analysis (to the basecalling task) has a great potential for eliminating low-quality and redundant nanopore reads that overwhelm downstream analysis (e.g., basecalling, variant calling, alignment) especially with large scale genomes. However, in the case of the HMM, the studies conducted in [13,14] have demonstrated a maximum power budget of 5 W and 6 W, respectively, and an accuracy of 98% as reported by Timp et al. in [10]. Hence, an essential trade-off for choosing between DNNs and DP approaches subject to the target application requirements and/or constraints is still an open problem. In this paper, we particularly focus on the HMM-based detection due to its applicability in various potential applications with a reasonable accuracy, albeit, enough to detect a bacterial E.Coli infection as shown in [15] in conjunction with its superior energy-efficiency that makes it a potential candidate for mobile sequencing platforms.

### 1.2. Motivation, Scope, and Contribution

In this paper, we extend the embedded vision previously presented in [16] to further demonstrate its effectiveness in addressing the mobile DNA base sequence detection problem. The proposed embedded framework integrates a custom RISC-V processor, optimized to execute a Viterbi–HMM basecaller, onto a Virtex-7 FPGA platform. Unlike the prior Rocket-based RISC-V implementation adopted in [16], the proposed design employs a proprietary TRV64P5 core with a tailored memory interface and tightly coupled instruction pipeline for improved performance-per-watt. To envision the upper bound computing requirements and match the conducted tests in [16], we sweep the complexity of the sequence detector, which would practically align with different targeted biomolecular sensing technologies. The proposed embedded detector is experimentally evaluated using the FPGA device and tested directly using a host PC via the PCIe serial interface. We benchmark the proposed system against x86, ARM Cortex-A9, Rocket (reported in [16]) implementations, and DL-based detectors, demonstrating its superior energy efficiency with an inference accuracy that meets the requirements for field-grade biosensing applications. In sum, this work advocates for a sensor-aware, resource-conscious basecalling paradigm in mobile DNA sequencing platforms—one where the power–performance–accuracy trade-off is holistically optimized for deployment at the edge of biosensing networks. The specific contributions of this work are summarized as follows:

- A novel energy-efficient Viterbi-based embedded sequence detector employing (for the first time) the proprietary Synopsis TRV 64-bit ASIP core is proposed for nanopore-based mobile DNA sequencing platforms.
- The TRV core-based sequence detector is realized on a Virtex-7 FPGA device with the aid of custom memory controllers for the TRV core architecture.
- The implemented FPGA design architecture is tested by stitching it to a commodity Intel CPU workstation over the PCIe 2.0 serial port. The communication and the flow control between the Virtex-7 TRV core and the CPU workstation is allowed via another custom state machine-based control interface for the RIFFA IP.
- For a varying nanopore *k*-mer base length of the sequence detector, the experimental results demonstrated a greater energy efficiency compared to state-of-the art Viterbi-based x86 and Cortex-A9 ARM implementations, as well as an existing Rocket core implementation by factors of 6.5×, 5.5×, and 4.6×, respectively. It also showed a better normalized performance by 1.8× and 2.4× compared to the Rocket and ARM detectors, respectively.
- The proposed detector is evaluated against recent DL-based basecalling implementations to underscore its superior energy efficiency, reinforcing its suitability for next-generation miniature nanopore sequencers.

## 2. Nanopore-Based Mobile DNA Sequencing

**Figure 1:**
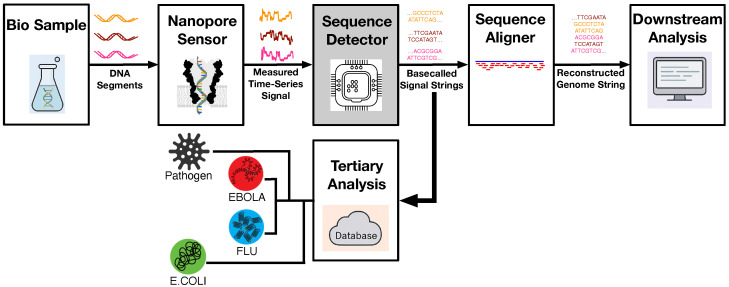
Nanopore-based DNA sequencing pipeline.

Nanopore-based DNA sequencing has emerged as a revolutionary technology, enabling long-read, label-free genome analyses in compact, portable platforms. Compared to traditional short-read methods, nanopore sequencing provides the ability to capture long DNA fragments directly from biological samples, making it ideal for rapid pathogen detection, metagenomics, and other field-deployable genomic applications. The complete workflow for the nanopore-based DNA sequencing pipeline can be illustrated as shown in Figure 1. The process begins with the acquisition and preparation of DNA samples, which are introduced to a nanopore sensor (that will be detailed in the following paragraphs) to measure the ionic current as DNA strands pass through. These raw, time-series signals are processed by the embedded sequence detector—or basecaller—which serves as the core focus of this work. The basecalled strings are then passed to the sequence aligner for genome reconstruction. In the final downstream analysis step, higher-level bioinformatics tasks, such as quality score adjustment, variant calling, and annotation, can be performed to provide deeper insights into the detected genome. Meanwhile, to enable rapid in-field diagnosis, a dedicated tertiary analysis stage is deployed to compare the detected basecalled sequences with a local pathogen database, ultimately facilitating the quick identification of bacterial (e.g., *E. coli*) and viral agents (e.g., flu, Ebola) directly on-site. The major objective of the proposed framework in this paper is to develop an energy-efficient sequence detection engine on a RISC-V embedded platform that allows such rapid identification, and making it practical for mobile DNA sequencing deployments. Such a rapid and low-power design empowers mobile genomic devices to operate effectively in remote or resource-constrained environments, making genomic diagnostics both accessible and actionable at the point of need.

**Figure 2:**
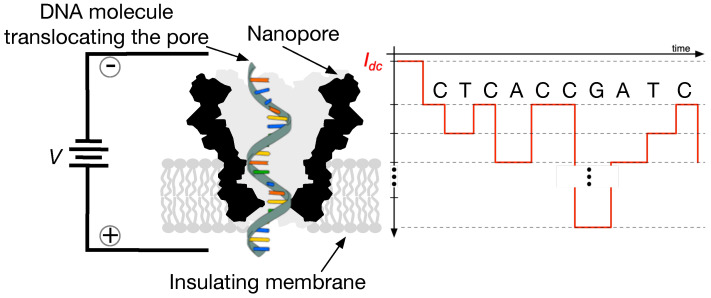
An example of a nanopore sensor interacting with a DNA strand.

**Figure 3:**
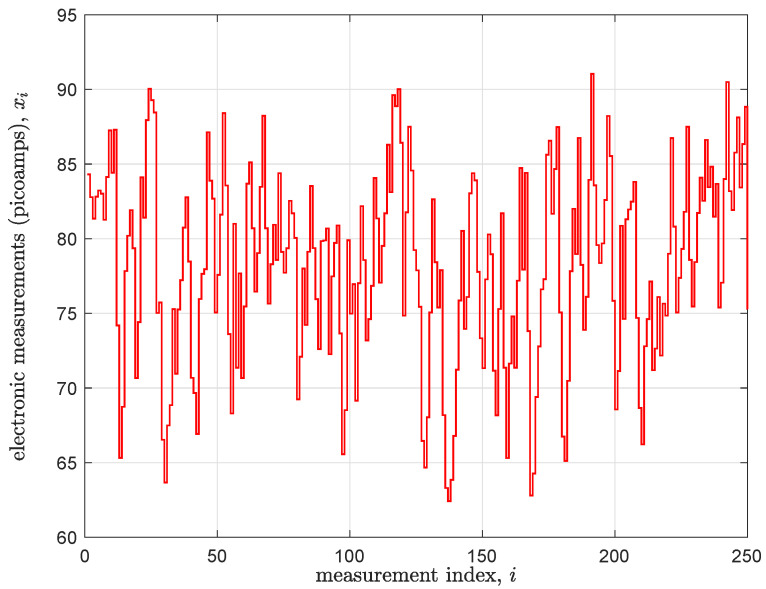
An example of a nanopore signal output; current fluctuations induced by the movement of DNA through the sensor.

As with many standard sensor technologies, nanopore sensors adopted in mobile DNA measurement machines are now capable of producing a constant stream of real-time data. An example depiction of such a sensor along with its means of operation is shown in Figure 2. The drawing shows a cutaway representation of a nanopore, which is a bio-engineered barrel-shaped protein. This molecule is placed in a conductive solution that induces an ionic current to flow through the sensor when a voltage is applied across it. When DNA strands are introduced into the solution, they eventually encounter a nanopore and also thread through its opening. Thus, as DNA passes through the nanopore, it fluctuates the pre-established current (pico-ampere to nano-ampere range) and thus generates signals of the type pictured in Figure 3. More details about nanopore sensors and their means of operation are provided in [21,22,23].

The measured signals are essentially real-time signatures of the DNA that passes through the nanopore. They can be used to identify the DNA’s structure. However, this raw information must be subjected to extensive bioinformatic signal processing in order to extract actionable information such as the identity of the organism whose genome samples passed through the device.

Such raw signals are produced by many DNA measurement channels working in parallel. Current technologies realize about 350 high-speed channels per cm^2^ and are packaged within a volume of about 100 cm^3^. Such metrics are already promising for wide deployment. With a broad development horizon left for this technology, it is conceivable that these properties will greatly improve in the years to come and thus present the possibility of multitudes of high-throughput molecular measurement machines distributed in the field. Naturally, this will create a significant challenge for the communication and analysis of the measurement results.

Given current designs, if all channels were simultaneously engaged in DNA measurements, the equivalent of one complete human genome could be measured by a 1 cm^2^ sensor array in five hours. Given that experiments have shown the ability of DNA to pass through such sensors roughly $10^{3}\times$ faster than currently realized, this means that such processing rates have the opportunity to improve profoundly.

In practice, existing portable measurement devices currently produce about 2.5 MB/s of raw data when fed with adequate amounts of source DNA. This is roughly the equivalent of three HD video services streaming in parallel. Transmitting such data continuously to remote bioinformatic processors for information extraction is conceivable, but certainly will come at a significant cost in power for any remotely positioned device. The ability for a DNA meter to enact its own local computations and only transmit digested data, at lower bandwidths, for ensuing processing in remote centers may be the foundation of a solution much more amenable to scaling.

The local computations desired of a DNA measurement device could be many and will be highly influenced by the information desired. Perhaps the most broadly applicable digest however is *base sequence detection*; this is the process of converting signals such as those pictured in Figure 3 into their text string equivalent. In particular, the process extracts a key structural feature of a DNA strand—its specific sequence of ‘base’ molecules drawn out of the discrete alphabet A={‘A’,‘C’,‘G’,‘T’} as shown in Figure 2—from the raw measurement signatures provided by the DNA nanopore sensor.

One algorithmic approach to DNA sequence detection, and the method pursued in this paper is discussed in Section 3 immediately below. It, as well as all other known approaches to this problem, is a computationally intensive affair. This is so given the relatively noisy state of the measured data (i.e., due to thermal variation in the nanopore sensor and its subsequent signal conditioning circuits) and the desire to minimize the number of errors in the called text output. Hence, detectors for contemporary mobile DNA measurement machines face a significant inference challenge in going from electronic measurements to text labels. Thus, for the prospect of implementing base detection as a native property of a DNA meter within a reasonable throughput and power budget, almost certainly some form of custom hardware will be necessary to enact the algorithm.

If adequately realized upon a dedicated hardware substrate, the base-sequence output of a detector, a ‘read’, could then be used for any of a broad variety of follow-up bioinformatic calculations. These would be intended to extract higher levels of insight. For example, a multitude of detector reads could be analyzed and used to infer the presence of certain genes, organisms, or other interesting biomarkers. This possible need for additional in situ bioinformatic processing, and even the typical preference of system designers’ for more flexibility (e.g., such as the need to deal with possibly hundreds of parallel measurement channels) suggest that any hardware solution should be effectively integrated within a more general, heterogeneous, computing system. Thus, we discuss a solution that employs an embedded processor for the task.

## 3. Viterbi-Based Sequence Detection

The conversion of a noisy time-series signal into a sequence of discrete states is a classic problem in digital signal processing and bioinformatics. A powerful and widely used tool for this task is the HMM, a statistical model that describes a system that transitions through a sequence of ’hidden’ states over time. In nanopore sequencing, the hidden state is the actual DNA k-mer (e.g., ’ACGT’) currently influencing the sensor. This state is not directly visible; instead, only a noisy sequence of “observations” is available—namely the measured ionic current values as depicted in Figure 3. The HMM mathematically connects these observations to the hidden states through emission probabilities (the likelihood of observing a certain current given a specific k-mer) and transition probabilities (the likelihood of moving from one k-mer to the next). Given a sequence of these noisy current measurements, the goal is to infer the most likely sequence of hidden k-mer states that generated them. The Viterbi algorithm is a highly efficient dynamic programming method designed for this purpose. It recursively computes, for each state and observation, the highest probability of any path ending in that state, then performs a traceback to reconstruct the optimal state sequence. Our proposed detector implements this Viterbi-based HMM decoding process directly in hardware for efficient, real-time nanopore basecalling.

As with many standard sensor devices, mobile DNA measurement machines are now capable of producing a constant stream of real-time data. The smallest of these machines are based on nanopore sensors [5]. For the purpose of DNA base detection, a ‘perfect’ nanopore sensor would emit one unique signal level for each of the four distinct nucleobases (*bases*) drawn from the set A={‘A’,‘C’,‘G’,‘T’} that comprise naturally occurring single-stranded DNA strings. In practice, nanopores output complex time-series signals exhibiting ${\left|A\right|}^{k}$ unique signal levels [10], one for each combination of four bases in a *k*-base-long (*k*-mer) sequence. The better the fidelity of a nanopore sensor the lower the *k* value (e.g., in [10] a 3-mer sensor is discussed).

A number of algorithmic options exist for the realization of sequence detectors. A prominent contemporary approach might invoke DL methods to do so. In this paper, we focus on a more established approach that may be of sufficient service for field devices with power and computational resources less amenable to the needs of today’s DL techniques. It is worth noting that field devices have emerged with the mobile sequencing market that was officially established in 2014 with the presentation of the first portable sequencer known as MinION [24]. Since then, mobile sequencers have been envisioned as a potential solution for field-based applications (e.g., bio-hazard monitoring [25], quick diagnosis by doctors at clinics [26], pathogen detection during pandemics) and IoT-based remote health monitoring [27]. Meanwhile, the recent study in [18] showed that the power consumption budget of DL-based detectors is not yet close to the target mW margin for battery-operated mobile sequencers. Hence, we consider the Viterbi algorithm for building our detector. This approach is pervasive in communication systems [28], but requires substantial customization for the base detection task.

In line with classical implementations of this algorithm, the Viterbi detector iteratively computes and keeps track of the probabilities of all possible base outputs over the course of *N* consecutive observations, also referred to as *events*. Typically, for each sensor, the detector will consider *N* observations, a number dependent on the length of the DNA strand to have traversed the pore, and from those observations call the base molecule sequence of the strand. For a system consisting of *P* pores, which encounter *K* strands per second on average where each strand has an average ‘length’ of *N* events, a (multiplexed) detector that processes P·K·N events per second is required.

**Figure 4:**
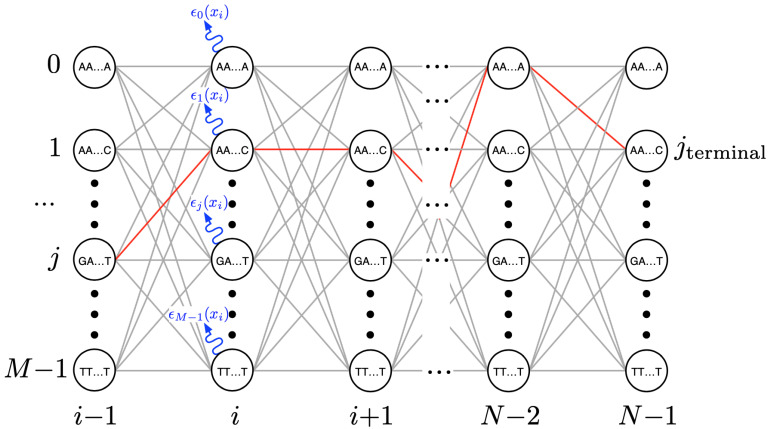
Generalized depiction of the signal trellis through which a Viterbi detector operates. Each state denotes the possible combination of bases in a *k*-mer.

A trellis diagram depiction of the algorithm’s transitions across the detector’s state space as indicative of its response to the signals coming from one pore operating on one DNA strand is shown in Figure 4. The circles represent the signal ‘states’ accounted for by the detector. For a detector that assumes sensor measurements are a result of *k*-mer excitation, a total of $M={\left|A\right|}^{k}$ states, j∈{0,…,M−1} must be accounted for at each new event observation *i* (up to *N* observations per strand). A number of parallel calculations, one per state, comprise the detector’s computational workload at each *i*.

To begin with, at each event *i*, the detector must compute the *emission* likelihood, ϵ (see Figure 4). This quantifies the likelihood that a newly arriving measurement signal level, $x_{i}$, may be associated with the detector’s sensor model parameters. This model consists of the set of expected signal levels $\left\{\mu_{j}\right\}$ for each possible state *j* as well as the extent to which a measurement may deviate from it, the set of standard deviations $\left\{\sigma_{j}\right\}$. Such models are typically learned as part of a training process (e.g., Baum–Welch algorithm) [29], and would be provided to any embedded bioinformatics engine. This aspect is, therefore, not discussed further in our paper.

Assuming statistics that adhere to Gaussian assumptions, the expression for the likelihood variable of each possible state at each observed event $x_{i}$ is then

(1) $$\epsilon_{j}\left(x_{i}\right)={\left(2\pi \sigma_{j}^{2}\right)}^{-\frac{1}{2}}\exp\left[{\left(x_{i}-\mu_{j}\right)}^{2}/2\sigma_{j}^{2}\right].$$

As part of the detector’s iterative advance through its sequence of observations, the detector then applied Bayes’ rule and updates its posterior probability approximation for each state *j* at time *i*, $\alpha_{i}\left(j\right)$, via the recurrence equation

(2) $$\alpha_{i}\left(j\right)=\epsilon_{j}\left(x_{i}\right)\max_{\nu \in \omega (j)}\left[\alpha_{i-1}\left(\nu \right)\tau \left(\nu ,j\right)\right].$$

That is, the posterior probability of state *j* after the *i*th event is the product of its emission likelihood, $\epsilon_{j}$, and the most likely prior, the ‘max’ term in (2).

As shown, the prior is computed by considering another state ν (drawn from a subset of the *M* possible states) and multiplying its posterior from the preceding observation, $\alpha_{i-1}\left(\nu \right)$, by τ(ν,j), the *transition* probability that the state ν could have possibly been observed before the state *j*. Out of *M* possible states that could in theory be observed before *j*, only a finite subset, ω(j), have a non-zero τ(ν,j) and hence only their contribution to the posterior is accounted for. In our case, to account for insertions and single deletions, we used |ω|=21. Finally, only the maximum posterior product is retained to arrive at $\alpha_{i}\left(j\right)$ as expressed in (2).

The sets ω(j) and the transition probabilities, τ(ν,j), associated with them essentially constitute another set of model parameters. As with the $\mu_{j}$ and $\sigma_{j}$ settings discussed above, these would also be the result of a training process. Among the critical measurement problems addressed by these transition settings is the problem of jitter inherent to this measurement scheme. Since the movement of DNA through the nanopore sensors is not yet robustly controlled, it is possible that some inputs $x_{i}$ relate to exactly the same *k*-mer that was measured at i−1 (i.e., a measurement *insertion*) or that a particular *k*-mer’s measurement is not registered at all (i.e., a measurement *deletion*). Expanding the size of ω gives the detector more flexibility in recognizing such states, with added computational cost of course. In our case, to account for insertions and single deletions, we used |ω|=21.

Equations (1) and (2) constitute the primary computational workload of the detector. Besides these probability calculations, however, we also need to compute sequences of pointers that will be used to traceback across the trellis, from the *N*’th observation to the first (e.g., red path in Figure 4), in the process of identifying the most likely measured DNA base sequence.

This pointer generation operation can be distilled to

(3) $$\beta_{i}\left(j\right)=\arg\max_{\nu \in \omega (j)}\left[\alpha_{i-1}\left(\nu \right)\tau \left(\nu ,j\right)\right]$$

which extracts the numerical label of the state selected to serve as the prior for the calculation of the posterior $\alpha_{i}\left(j\right)$.

After processing the last event $x_{N-1}$, the detector simply fetches the most likely terminal state according to

(4) $$j_{\mathrm{terminal}}=\arg\max_{j}\left[\alpha_{N-1}\left(j\right)\right]$$

and then recursively executes the traceback kernel of its algorithm to traverse the previously computed sequence of pointers from that location, $\beta_{N-1}\left(j_{\mathrm{terminal}}\right)$, to the beginning of the measurement. The completion of this step concludes the detector’s job for one strand through one sensor.

## 4. Proposed Embedded Detector

In this section, we present our proposed embedded implementation of the Viterbi detector, discussed in Section 3, on a RISC-V (RV) microprocessor core. We utilize the Synopsys’ TRV cores family of application-specific instruction-set processors (ASIPs) [30] to implement a 64-bit RV instruction set architecture (ISA) core with five pipeline stages (i.e., known as TRV64P5 core). Without loss of generality, the RV core is then realized on a Virtex-7 FPGA device to run our detection program and measure its energy efficiency.

### 4.1. RISC-V Architecture Description

**Figure 5:**
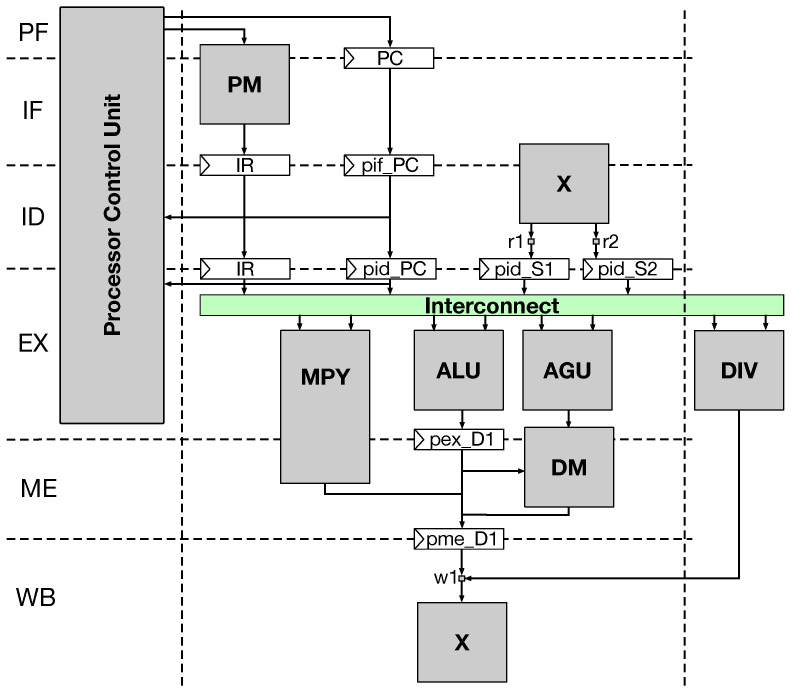
The TRV64P5 core’s micro-architecture.

The five pipeline stages of the adopted RV architecture can be understood in light of the core micro-architecture shown in Figure 5. The first stage in the pipeline, the *instruction fetch* stage (IF), moves each new 4-byte instruction from the core’s program memory (PM) into an instruction register (IR). The second pipeline stage is the *instruction decode* (ID) which extracts the instruction operands and directs them to the appropriate functional unit (i.e., arithmetic logic unit (ALU), address generation unit (AGU), multiplication unit (MPY), and division unit (DIV)) from its 64-bit central register file (X). The *instruction execution* (EX) comes in the third stage of the pipeline during which functional units execute their operations, and the load/store operations from/to the data memory (DM) is initiated (i.e., after the AGU memory address computations takes place). The fourth stage in the pipeline is reserved for *memory access* (ME) where a load operation’s result is sent to the data bus and the data to store are sent to the memory. The fifth stage is *write back* (WB), which is responsible for writing the execution results computed by the core’s functional units (during the EX stage) to their destination fields in the central register file. It can also be noted that the block diagram depicted in Figure 5 shows an additional pre-fetch stage (PF) implemented in the processor control unit (PCU). The PF stage handles the next program counter (PC) computation (i.e., program address for an instruction) using the 64-bits pre-fetch register PC just like the instruction caching in traditional architectures.

The block diagram shows also the pipeline registers at each stage, which starts with the letter p (e.g., pid is the pipeline register written in the ID stage). The central register file, denoted as X, has two read ports r1 and r2 and one write port w1. It can be also noted from Figure 5 that the MPY is a 2-stage multiplier unit which starts in the EX stage and finishes its execution in the ME stage. Finally, the DIV unit is an out-of-pipe multi-cycle divider unit which starts in the EX stage and can take an arbitrary number of cycles to finish.

### 4.2. Architecture of the Proposed RV-Detector

**Figure 6:**
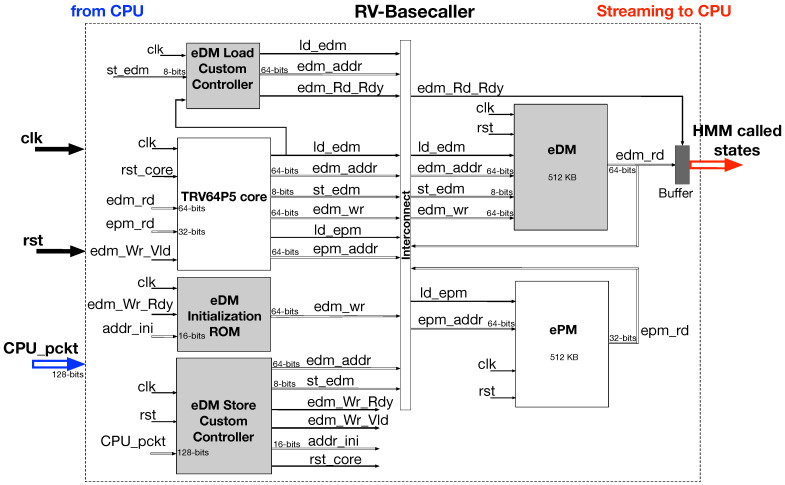
FPGA system architecture for the TRV64P5 core-based detector.

The system architecture of the proposed RV-detector is shown in Figure 6. To maintain its operation, the TRV core accesses two external memories that directly support its internal program and data memories. In particular, the 512 KB external program memory (ePM) is pre-initialized with the entire detector program. The 512 KB external data memory (eDM), on the other hand, is initialized with the Viterbi model parameters at the beginning of the core operation and also used for external storage during the program execution.

The initialization procedure of the eDM is carried out using a read-only memory (ROM) and under the supervision of the eDM *store custom controller*. The eDM initialization takes place at the beginning of the core’s operation (i.e., processing a single event file which corresponds to one strand measurement by the nanopore) upon receiving a control packet from a separate host CPU. More details about the actual implementation and testing setup for the detector are provided in the following subsection.

After eDM initialization, the eDM’s store custom controller de-asserts the core’s reset allowing it to start loading the program from the ePM for execution. Following the core’s last execution cycle, the eDM becomes solely managed by the eDM *load custom controller*. The load custom controller is used to initiate the loading process for the detection results (i.e., states’ sequence) from their designated address locations inside the eDM. The eDM loaded results are then streamed out to a host CPU as illustrated in the following subsection.

### 4.3. Hardware Realization/Testing Framework for the Proposed RV-Detector

**Figure 7:**
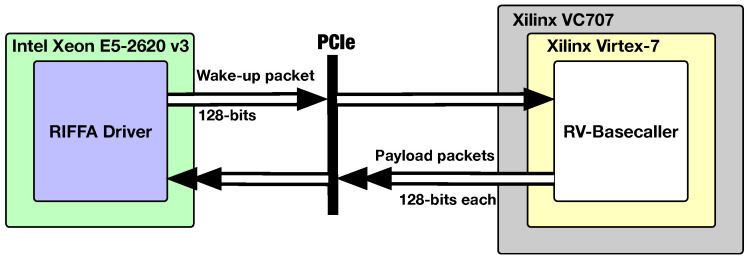
FPGA realization and testing platform for the RV-detector.

In order to experimentally test our RV-detector using the RISC-V architecture explained above, we utilize a CPU-FPGA serial communication platform as illustrated in Figure 7 to realize it. The platform setup is composed of a host Intel Xeon E5 CPU, PCIe 2.0 interface, reusable integration framework for FPGA accelerators (RIFFA) [31], Xilinx VC707 evaluation board hosting Virtex-7 FPGA device. It should be noted that our RV-detector is fully realized on the Virtex-7 device as explained in Figure 6. The host CPU is only used to control the detector’s operation and analyze its results. The detector results are streamed out from the FPGA, via the PCIe serial interface, to the CPU in 128-bit RIFFA packets.

**Figure 8:**
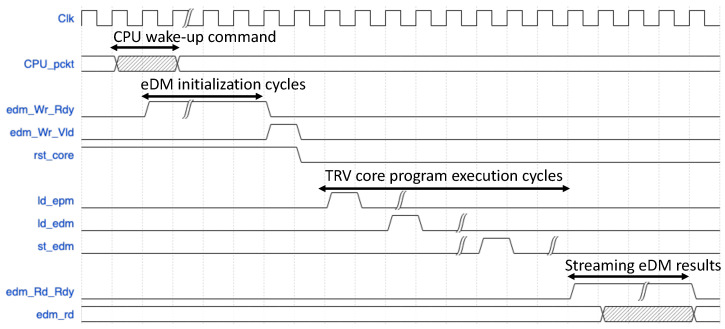
Timing waveforms for the RV-detector.

The flow control mechanism for the operation of the testing platform depicted in Figure 7 can be illustrated using the timing diagram in Figure 8. The host CPU first sends a 128-bit wake-up packet to the RV-detector. Upon receiving the wake-up packet from the CPU, the RV-detector starts by initializing its eDM. After the memory initialization cycles are fully consumed, the eDM store custom controller commands the RV core to start the execution of the ePM’s detection program. After spanning the program execution cycles, the RV core stores the final called state sequence inside the eDM in a certain address range. The eDM load controller starts loading the called states from the eDM and sends a ready signal (i.e., edm_Rd_Rdy) for the Tx engine of the RIFFA IP (i.e., wrapping the RV core). The RIFFA Tx engine consequently streams the loaded results to the host CPU (for verification) via the PCIe bus in 128-bit payload packets.

## 5. Experimental Results

In this section, we present a comprehensive experimental evaluation of our proposed single-core RISC-V detector. To ensure fairness and clarity, the evaluation is conducted in two distinct stages.

First, we compare our detector against other classical hardware platforms—an x86 CPU, an ARM Cortex-A9 processor, and a RISC-V Rocket core—running the identical Viterbi-based detection approach. For this stage, we maintained identical experimental conditions: the same Viterbi–HMM sequence detection program and the same input event sequence lengths were used across all hardware platforms. The input data were generated using k-mer signal models provided by Oxford Nanopore Technologies (ONT) [32], which are widely accepted in the nanopore community for simulating realistic signal characteristics for benchmarking purposes. The results for the Rocket core are adopted from our prior work [16]. This direct comparison methodology allows us to isolate and fairly evaluate the impact of the underlying hardware architecture on performance and energy efficiency for the exact same computational task. It is also worth noting that the x86 CPU is the 12-core Intel Xeon E5-2620 v3 processor clocked at 2.4 GHz with 32 GB of RAM. The ARM CPU is the dual-core Cortex-A9 MPCore hosted on the Zedboard Zynq-7000 ARM/FPGA SoC development board that is clocked at 667 MHz with 32 KB L1-cache, 512 KB L2-cache, and 256 KB on-chip memory. The Rocket design adopts a Spartan-6 FPGA device clocked at 50 MHz.

Second, we extend our comparisons to evaluate the effectiveness of our design against two state-of-the-art deep learning (DL)-based basecallers, DeepNano-Coral and Guppy, as reported in [18]. For this analysis, we cite the evaluation metrics (performance, active power, and accuracy) from the peer-reviewed study by Perešíni et al. Consequently, the hardware configurations (which involve high-performance x86 CPUs and GPUs), input datasets (which included real-world Klebsiella pneumoniae and human genomic reads), and sequence lengths used in their evaluation were not identical to our own. The goal of this second comparison is not a fine-grained benchmark, but rather a system-level evaluation of the broader trade-offs between our classical probabilistic approach and the DL paradigm. This highlights the practical implications of deploying a low-power, resource-efficient solution versus a high-throughput, power-intensive one in the context of mobile, field-deployable DNA sequencing.

### 5.1. Evaluation Against HMM Detectors

The evaluation considers a series of detectors with exponentially growing complexity demands: 3-mer (64 HMM-states), 4-mer (256 HMM-states), and 5-mer (1024 HMM-states). In each case, the same program runs on the x86 CPU, the VC707-enabled system architecture proposed in Figure 7, the ARM CPU, and the Rocket CPU. To further ensure fair comparison with our single-core RV-detector, similar to that presented in [33], the program is executed on a single core for both the x86 and the ARM processors. Also, PAPI timers [34] were used to measure the cycles count of the x86 CPU that corresponds to the program execution to allow fair comparison with the bare metal implementations on the RV, ARM, and Rocket cores.

**Figure 9:**
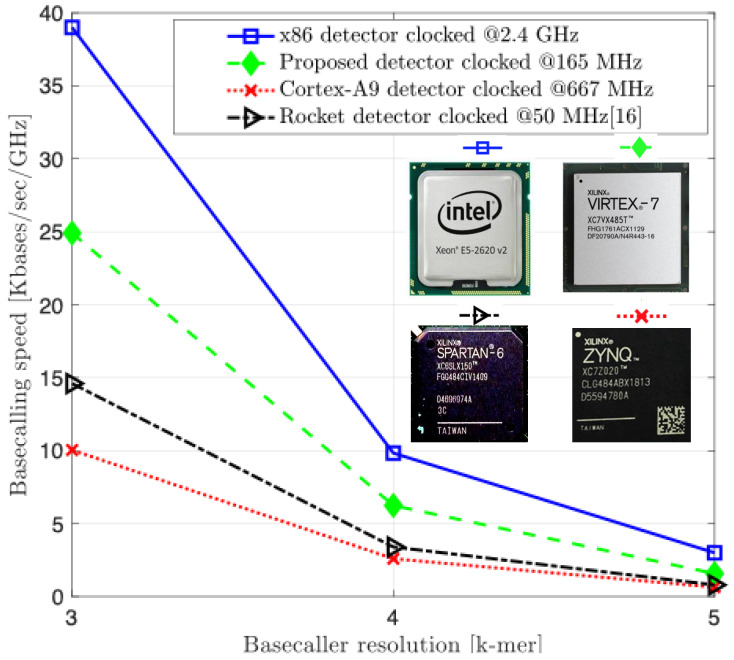
Experimental detection speed.

The results depicted in Figure 9 highlight the measured performance of our proposed RV-detector compared to the aforementioned implementations. The speeds are presented in the number of kilobases (Kbases) processed per second per GHz of the operating clock. The normalization to the clock frequency is to better understand the performance comparison of the four systems while being unavoidably clocked at different rates. Not surprisingly, the speed measurements show that the x86 CPU has generally outperformed other implementations for the three detector versions. Likely this result pertains most directly to the reference computer’s bigger memory resources, out-of-order architecture, and higher available power.

**Figure 10:**
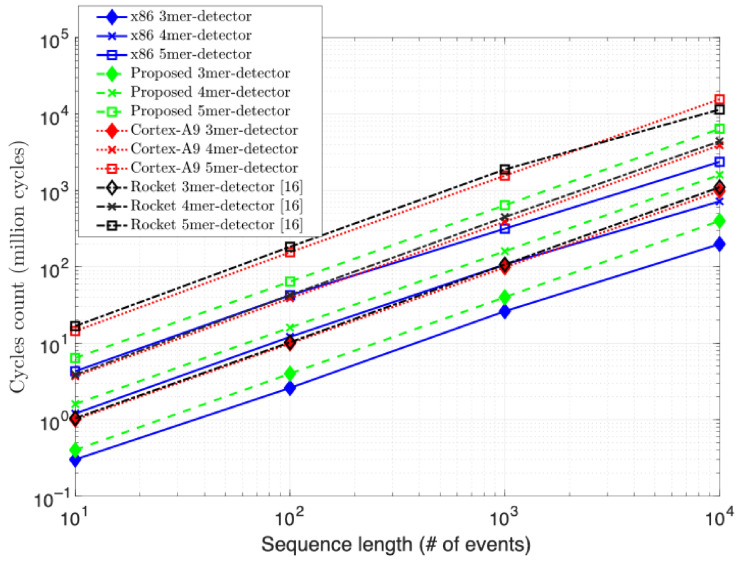
Experimental cycles count.

As the complexity of the detection problem increases, the performance of all designs drops off in proportion, by about a factor of four at each increment. Only the x86 CPU’s drop off is more graceful between the 4-mer and 5-mer increment by a factor of 3.2×. This is likely not only a testament to the reference computer’s greater power, clock, and memory resources, but also its sophisticated architecture. For example, the Intel design is a super-scalar design with speculation, and thus, also a richer target for compiler optimizations. In contrast, the RV, ARM, and Rocket are a single-issue architectures with no out-of-order processing capability. In addition, the cycles count comparison in Figure 10 further supports the normalization introduced to the performance results of Figure 9. The proposed RV-detector’s performance consistently stands right below the x86 and above the other two counterpart implementations, as in Figure 9, for the three addressed complexities when increasing the input sequence length.

**Figure 11:**
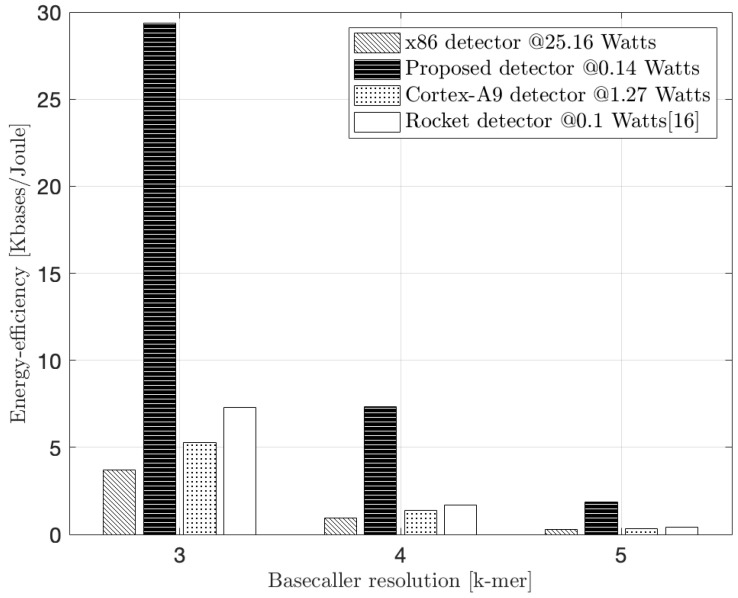
Experimental energy efficiency.

In Figure 11 we present the measured energy efficiency of our design. This refers to the the Kilobases processed per Joule of energy consumption. For the x86 CPU, Virtex-7, Zynq-7000, and Spartan-6 FPGAs, these measurements compare the extra energy needed to process the detection algorithm alone and do not account for ancillary power that is consumed with the device operating in the quiescent state (i.e., 62 W for the x86 CPU, 3.6 W for the Virtex-7 FPGA, 2.5 W for the Zynq-7000 FPGA, and 0.2 W for the Spartan-6 FPGA as reported in [16]). During program execution, the x86 CPU consumes an extra 25.16 W while our proposed RV core, the ARM processor, and the Rocket processor of [16] drain 140 mW, 1.27 W, and 100 mW, respectively.

As a result of the low energy drain, our RV embedded system shows an improvement factor of 8×, 6× and 4× over the x86 CPU, the ARM CPU, and the Rocket design in [16], respectively, at 3-mer detection complexity. It also should be noted that our embedded Virtex-7 FPGA design is realized in a 28 nm technology and operates from a 1.8 V supply while our reference Intel processor is implemented in a 22 nm technology and includes sophisticated power management within a window of 0.65–1.3 V. This endows the CPU with a 3× power advantage relative to the FPGA, a disparity not normalized for in Figure 11. On a more even footing, it is likely that the energy efficiency advantage for the specialized core at this complexity would be even greater. Unlike the ARM CPU and the Rocket design of [16], our RV core efficiency advantage slightly declines at greater algorithm complexity compared to the x86 CPU (i.e., 6.5× at 5-mer complexity), a result of the more sophisticated Intel processor’s ability to exploit its inherent structural parallelism and speculative execution sequencing. In particular, the shrinking in the energy efficiency at k-mer = 5 is a natural result due to the increased search space of the corresponding nanopore state for each new input event to 1024 states (i.e., 4× bigger than the 4-mer case and 16× bigger than the 3-mer case). Consequently, the latency of constructing the states’ trellis across the input event sequence becomes significantly bigger compared to 3-mer and 4-mer nanopore cases. That increase in latency results in a drop in the detector’s performance, and hence, the energy efficiency. Nevertheless, the proposed detector demonstrated a normalized performance improvement of approximately 2× and 3×, and an energy efficiency improvement of 5× and 6× over the Rocket and Cortex-A9 detectors, respectively, at k-mer = 5. This directly results in a relatively longer battery life-time for a mobile sequencer equipped with the proposed detector, compared to the Rocket and ARM detectors, for the same sequencing application.

To further clarify the dominance of the proposed detector over the ARM and Rocket detectors in terms of performance and energy efficiency (i.e., energy efficiency = performance (in bases-per-second)/power consumption (in watts)), a break down analysis for the run-time cycles and the power consumption is provided in Table 1. The analysis shows that the major part (97% on average) of the sequence detection algorithm workload (measured in run-time cycles) is consumed by the trellis kernel for all implementations. The remaining run-time cycles is totally consumed by the traceback kernel in case of the proposed detector, whereas in the case of ARM and Rocket detectors a portion of the remaining cycles is consumed by the off-chip communication with the DRAM as a result of the cache memory misses (i.e., L1-cache in case of the Rocket detector, and L2-cache in case of the ARM detector). For the Rocket detector, the additional DRAM access cycles results in an estimated power budget of 7 mW on top of the 93 mW core power. To ensure an accurate breakdown for the Rocket’s power budget, the power consumption ratio for the core processing cycles to the DRAM access cycles is estimated jointly based on the area report (i.e., ratio of 13:1, respectively) and the run-time cycles ratio for the core versus the DRAM communication link. In the case of ARM, there is no available on-board facility (the DRAM is part of a ZedBoard FPGA evaluation system) to actually measure the power consumption for the Zynq-7000 FPGA to communicate with the DRAM during operation. However, the 1.27 W power budget for the ARM core is clearly overwhelming the mW budget of the proposed and Rocket detectors. In sum, the performance and energy efficiency superiority of the proposed detector pertains exclusively to its adopted TRV core simple ISA, which requires substantially lower instruction cycles with its on-chip memory structures (eDM and ePM) compared to the ARM and Rocket architectures within a conservative power margin (i.e., comparable to Rocket and substantially lower than ARM).

### 5.2. Evaluation Against DL Detectors

Table 2 presents a comparative evaluation between the proposed embedded Viterbi–HMM sequence detector and two widely adopted DL-based basecallers: DeepNano-Coral and Guppy reported in [18]. It is important to note that all measurements for Guppy and DeepNano-Coral illustrated in Table 2 are cited directly from [18]. These comparisons underscore the practical advantages of the proposed detector in mobile DNA sequencing platforms, especially for field-deployable applications where power and energy constraints dominate system design. While DL-based basecallers generally achieve higher sequence detection accuracy, this improvement comes at the cost of significantly increased power and resource demands. Both Guppy and DeepNano-Coral require high-performance x86-class systems accelerated with discrete GPUs (NVIDIA GTX 1650), drawing 48 W and 92 W of active power, respectively—excluding idle baseline power. In contrast, the proposed detector achieves an energy-efficient operation at just 0.14 W, delivering 29 K bases/Joule compared to only 12 K and 2 K bases/Joule for Guppy and DeepNano-Coral, respectively.

The performance gap—4 K vs. 223 K–553 K bases/s—is largely attributed to the GPU-accelerated multi-core CPUs used in DL detectors, which are likely clocked at significantly higher frequencies than the 165 MHz Rocket core implemented on our unaccelerated FPGA platform. Notably, our design refrains from GPU or hardware accelerator support by intent, maintaining fidelity to the low-power, lightweight constraints of mobile DNA sequencers. Despite the reduced throughput, the proposed detector can still process a full viral or bacterial genome ranging from 10 K to 100 K bases (e.g., Influenza A (≈13.5 Kb), Ebola (≈19 Kb), and SARS-CoV-2 (≈30 Kb)) in 2.5 to 25 s, which is sufficiently fast for real-time field diagnostics and outbreak response.

Moreover, the 90% detection accuracy achieved by the proposed embedded detector (i.e., the 3-mer nanopore case as detailed below in Section 5.3) is sufficiently reliable for downstream tertiary analysis, including the identification of pathogens such as *E. coli*, flu, or Ebola, as depicted in the mobile sequencing pipeline in Figure 1. This level of fidelity enables practical, on-device genomic screening, particularly in scenarios where full genome assembly is not a prerequisite. It is worth noting that the detection accuracy was evaluated using nanopore k-mer signal models provided directly by ONT [32]. These predictive models characterize the expected current response for each k-mer under defined sequencing conditions and are specifically intended for developmental use. Their fidelity and representativeness make them highly suitable for simulating realistic nanopore signal behavior and validating embedded sequence detectors.

It is also worth noting that while recent advancements in efficient deep learning [35] (e.g., quantized neural networks, lightweight CNNs, and transformer compression strategies) are promising for edge inference, their application to nanopore basecalling remains limited. These models typically require dedicated AI accelerators and power envelopes in the 1–10 W range [36]. In contrast, our proposed Viterbi–HMM solution operates at just 140 mW, using only general-purpose logic on a RISC-V core. It also avoids the need for large training datasets or specialized MAC hardware. This makes our approach particularly well suited for ultra-low-power mobile biosensing applications where sub-watt operation, predictable latency, and compact silicon area are hard constraints.

### 5.3. Accuracy Evaluation

To further clarify and quantitatively evaluate the accuracy trade-offs between HMM complexities, we conducted a detailed statistical analysis of the 3-mer, 4-mer, and 5-mer detectors. The simulation methodology was designed to mirror the operational conditions of our resource-constrained mobile platform. We performed 100 independent simulations for each model, averaging the results to ensure statistical stability. In each run, a new 1800-base random DNA sequence was generated, and the corresponding nanopore signal was simulated at a Signal-to-Noise Ratio (SNR) of 32 dB. Critically, this signal was processed in independent 384-event chunks to reflect the frequent ’cold starts’ imposed by a limited memory buffer. The average measured accuracy for the 3-mer, 4-mer, and 5-mer detectors is 90%, 92.93%, and 94.44%, respectively. The results demonstrate a clear trend of diminishing returns as model complexity increases.

The accuracy analysis provides the basis for a holistic design evaluation. While the 4-mer and 5-mer models provide progressively higher accuracy, this comes at the expense of exponentially increasing computational demand, which, as shown in Figure 9 and Figure 11, results in lower throughput and energy efficiency. For mobile, battery-powered sequencing applications where energy is the primary constraint, a modest gain in accuracy may not justify a significant reduction in battery life or processing speed. Hence, we identify the 3-mer HMM detector as an optimal design point for our target application. It achieves an accuracy level sufficient for many field-based screening tasks (e.g., pathogen identification) while offering the highest performance-per-watt, making it the most suitable choice for deployment in truly portable, energy-constrained biosensing systems.

In summary, the proposed Viterbi–HMM basecaller provides a practical balance between inference accuracy and energy efficiency, demonstrating its potential as a core computing engine in future mobile DNA sequencing systems designed for point-of-care or remote genomic surveillance.

### 5.4. Design Scalability and Practical Feasibility

Although this study focuses on single-channel performance evaluation, the proposed RISC-V-based detector is inherently extensible to multi-channel nanopore sequencing scenarios. Two primary design strategies are feasible: (1) multi-core instantiation, where multiple TRV64P5 RISC-V cores decode independent channels in parallel, coordinated by a data switching and aggregation interface; and (2) time-multiplexed execution, where a single core interleaves decoding across multiple channels. The former offers high throughput scalability at the cost of increased area and energy, while the latter favors compact, energy-aware deployments with modest performance requirements. These extensions can be tailored to application-specific constraints, making the proposed architecture a viable foundation for building scalable basecalling systems in mobile nanopore sequencing platforms.

Finally, we note that the proposed detector architecture is well suited for practical deployment in mobile sequencing platforms. Its ultra-low power (140 mW active), compact memory footprint (∼1 MB), and modular design make it amenable to SoC integration with passive cooling and minimal thermal constraints. The detector’s chunk-based processing allows flexible memory scaling, and further simplifications (e.g., offloading traceback) can reduce area and power for the most constrained applications. We envision this detector operating as a specialized co-processor in a mobile SoC that integrates analog sensing, RISC-V-based processing, and wireless communication, paving the way for future deployment in handheld, battery-operated, and even wearable biosensing systems.

## 6. Conclusions

This paper reported an embedded solution, based on the emerging RISC-V processor architecture, for practically running a critical bioinformatic machine learning algorithm in mobile DNA sequencers. A problem with substantial computing needs, but severe energy constraints. The proposed RV sequence detector was physically realized on a Virtex-7 FPGA device and tested using an x86-based workstation via a RIFFA-enabled PCIe communication interface. By comparing to a classical x86 CPU implementation, ARM-based SOC implementation, and another existing Rocket detector, the proposed detector was evaluated over a range of different complexities for its underlying Viterbi algorithm. The experimental results demonstrated the potential of the proposed detector with an energy efficiency improvement factor of at least 6.5× compared to a sophisticated 12-core out-of-order x86 superscalar processor, 5.5× to the Cortex-A9 ARM processor, and 4.6× to an existing Rocket detector. Despite the higher performance of the x86 processor relative to our single-core RV microprocessor, the energy efficiency superiority of our proposed design emphasizes its effectiveness for adoption in mobile DNA sequencers.

From another perspective, the comparative evaluation of the proposed embedded Viterbi–HMM detector against state-of-the-art DL-based basecallers showed 15× and 2.5× higher energy efficiency in favor of our proposed detector, while maintaining a competitive detection accuracy suitable for mobile DNA sequencing. Despite lower throughput, the detector can process typical viral or bacterial genomes within seconds, making it a practical solution for real-time, in-field pathogen identification. These findings reinforce the viability of our low-power basecalling framework for next-generation portable sequencing applications.

While the results presented in this work demonstrate the technical feasibility and energy efficiency of our proposed Viterbi–HMM-based RISC-V detector, several opportunities remain for further enhancement. First, the current FPGA-based prototype has been validated under a single-channel nanopore sequencing scenario. Although the architecture is inherently modular and readily scalable, experimental verification of multi-channel operation will be a key step toward enabling high-throughput mobile sequencing systems. Second, the algorithmic scope of the present design is centered on efficient basecalling for canonical nucleotide sequences. Extending the model to address advanced genomic features (e.g., base modification detection due to methylation and accurate handling of long homopolymers) represents an important research direction, particularly in domains where specialized DL-based methods have shown potential. Finally, while the FPGA implementation provides a cycle-accurate proof-of-concept, transitioning to an ASIC or SoC may yield different absolute performance and power characteristics due to technology-specific factors. All these opportunities form a clear roadmap toward realizing an ultra-low-power, field-ready mobile sequencing platform.

## Figures and Tables

**Table 1:** Performance and power breakdown comparison.

Platform	Trellis Cycles (%)	Traceback Cycles (%)	Off-Chip Comms Cycles (%)	Core Power (mW)	Off-Chip Comms Power (mW)	Total Power (mW)
Proposed detector	97	3	0	140	0	140
Cortex-A9 detector	98	1	1	-	-	1270
Rocket detector [16]	95	1	4	93	7	100

**Table 2:** Comparison with state-of-the-art DL-based detectors.

Platform	Performance (Bases/s)	Active Power (Watt)	Energy Efficiency (Bases/Joule)	Detection Accuracy (%)
Proposed detector (FPGA)	4K	0.14	29K	90%
DeepNano-coral (CPU + GPU) [18]	223K	92	2K	92%
Guppy (CPU + GPU) [18]	553K	48	12K	92%

## Data Availability

All relevant data are contained within the paper.
